# Oncological and Functional Outcomes After Minimally Invasive Surgery for Mid and Low Rectal Adenocarcinoma: A Review

**DOI:** 10.7759/cureus.82238

**Published:** 2025-04-14

**Authors:** Antonio Costanzo, Lorenzo Vescovi, Valentina Rampulla, Michela Caprioli, Michele Marini, Andrea Rigamonti, Daniele Passannanti, Valentina Crisafulli, Antonio Floridi

**Affiliations:** 1 General and Emergency Surgery, Azienda Socio Sanitaria Territoriale (ASST) Bergamo Est, Seriate, ITA; 2 General Surgery, Azienda Socio Sanitaria Territoriale (ASST) Bergamo Ovest, Ospedale di Treviglio-Caravaggio, Treviglio, ITA; 3 Pathology, Azienda Socio Sanitaria Territoriale (ASST) Bergamo Est, Seriate, ITA

**Keywords:** functional outcomes, mesorectum, minimally invasive surgery, oncological outcomes, rectal adenocarcinoma

## Abstract

In this study, we analyze the oncological and functional outcomes after minimally invasive surgery (laparoscopic and robotic) for mid and low rectal adenocarcinoma. This is a narrative review of articles published from January 2019 to December 2024 in which we analyzed the rate of short-term oncological outcomes (quality of surgical samples), long-term oncological outcomes (recurrence rate, overall survival, and disease-free survival), and functional disorders (urinary, sexual, and bowel function) after minimally invasive surgery.

The rates of complete mesorectum are 67.7%-92.8%, the rate of free circumferential resection margin is 94%-98.2%, and the rate of free distal resection margin is 99.4%-100%. The local recurrence rate is 2.3%-7.3%, the overall survival rate is 80%-95.6%, and the disease-free survival rate is 70%-86.4%. The rate of urinary disorders is 25%-26.5%, sexual disorders are 35%-80%, and bowel disorders are 17%-44.6%.

This review demonstrates that minimally invasive surgery yields favorable oncological and functional outcomes. The continuous evolution in robotic surgery will increasingly lead to interesting implications for rectal surgery, thanks to less surgical trauma and better intraoperative visualization of nerves.

## Introduction and background

Total mesorectal excision (TME) is the standard surgical approach for patients with mid and low rectal adenocarcinoma. It is based on a precise dissection in the avascular plane between the presacral and mesorectal fascia with the excision of the rectum along with surrounding lymphatic and vascular drainage. Quirke et al. described three grades of mesorectal quality obtained with surgery: mesorectal (complete), intramesorectal (nearly complete), and muscularis propria (incomplete) [1]. This aspect is very important because mesorectal quality is associated with the rate of local recurrence [2].

The evolution of laparoscopy has allowed the use of minimally invasive procedures in an increasing number of complex surgical operations. This approach offers several advantages such as early mobilization, shorter length of stay, earlier recovery of normal function, less postoperative pain, and a better cosmetic result than open surgery [3].

Recently, robotic surgery has been used as an option for rectal surgery. This approach has the advantage that delicate dissection can be performed in the narrow pelvic cavity using multiple joints. Some studies have reported that the oncological outcomes are similar to laparoscopy, but the functional outcomes seem to be better [4].

The oncological and functional outcomes are the subject of many studies in the literature because they influence the survival of patients and their quality of life. It is very important to inform patients about these aspects throughout the treatment process, whether they undergo neoadjuvant therapy or go directly to surgery.

In this review, we analyzed the short-term oncological outcomes represented by the quality of surgical samples with resection margins, and long-term oncological outcomes represented by recurrence rate, overall survival, and disease-free survival after minimally invasive surgery for mid and low rectal adenocarcinoma. Additionally, we analyzed the functional outcomes represented by urinary, sexual, and bowel function after rectal surgery.

## Review

Methods

We included articles published in English that analyzed oncological and functional outcomes of patients with mid and low stage I-III rectal cancer who underwent TME with minimally invasive surgery (laparoscopic and robotic) from January 2019 to December 2024. All studies were found by searching the PubMed and Embase databases. We included both patients who underwent chemoradiotherapy and those who did not; we did not include cases of lateral lymph node dissection.

Inclusion Criteria

Prospective, retrospective, and case-control studies of patients with mid and low rectal adenocarcinoma were included.

Exclusion Criteria

Non-English articles, case reports, reviews, unspecified histology, or cancers other than adenocarcinoma (rectal gastrointestinal stromal tumor (GIST), rectal neuroendocrine tumor, rectal sarcoma), and studies about only open surgery were excluded. 

After an initial search that yielded 113 results, we screened these articles by reviewing titles, authors, and abstracts. Ninety studies were excluded due to duplicates and treatment of other types of rectal cancer. Upon reviewing the full articles, we excluded 13 articles that did not meet the inclusion criteria: reviews and studies with unspecified histology. The final analysis was conducted on a total of 10 articles. The characteristics of the studies are reported in Table 1.

**Table 1:** Characteristics of the studies

Authors	Year of publication	Type of study	Number of patients	Laparoscopic/robotic
Pla-Martì et al. [5]	2021	Retrospective	311	108/0; others open
Collin et al. [6]	2024	Retrospective	1,635	1,350, unspecified ratio; others open
Bjoern et al. [7]	2019	Retrospective	85	85/0
Park et al. [8]	2021	Retrospective	134	67/67
Pennings et al. [9]	2024	Retrospective	1,294	Unspecified ratio
Donovan et al. [10]	2024	Prospective	100	100/0
Peltrini et al. [11]	2022	Retrospective	367	241/4; others open
Francis et al. [12]	2023	Retrospective	478	478/0
Shin et al. [13]	2021	Retrospective	313	166, unspecified ratio; others open
Jiang et al. [14]	2022	Prospective	1,039	685/0; others open

Results

Quality of Mesorectum

Quality of the mesorectum was classified according to Quirke grades as complete, nearly complete, and incomplete. Complete mesorectum is intact or with only minor irregularities on its smooth surface (< 5mm). Nearly complete mesorectum has slight irregularities on its surface (> 5mm) without exposure of the muscularis propria.

The incomplete mesorectum has defects with visible muscularis propria. Six studies in our analysis reported details about the quality of the mesorectum. One study described 83.8% complete and 16.2% incomplete mesorectum [5]. Three studies reported surgical samples with three grades of mesorectum: 75%, 66.7%, and 79.1% complete; 17%, 11.1%, and 9% nearly complete; 8%, 22.2%, and 11.9% incomplete [6-8]. Two studies reported only the percentage of complete mesorectum (92.8% and 86.8%) [9,10]. Four studies did not report data about the quality of the mesorectum [11-14].

Circumferential Resection Margin (CRM)

The CRM was considered positive when the distance between the surgical resection plane and the deepest cancer invasion was ≤ 1mm. Four studies did not report data about CRM [6,11-13]; the other six studies reported a percentage of negative CRM between 94% and 98.2% [5,7-10,14].

Distal Resection Margin

The distal resection margin is considered negative when the distance between the cancer and the resection margin is >1cm. Five studies did not report data about the distal resection margin [6,7,9,11,13], while the other five studies reported a rate of free margin between 99.4% and 100% [5,8,10,12,14].

Local Recurrence Rate

Local recurrence was defined as an intrapelvic tumor growth after surgery. Five studies did not report data about local recurrence [7-9,13,14]; the other four studies reported a rate of local recurrence between 2.3% and 7.3% [5,10-12]. Collin et al. [6] specified three different local recurrence rates based on the quality of the mesorectum: 2.3% for complete, 3.4% for nearly complete, and 6.9% for incomplete.

Overall Survival Ranging From Three to Five Years

Overall survival was defined as the time from rectal surgery to death from any cause. Our analysis included studies with overall survival data ranging from three to five years for patients who underwent upfront surgery or surgery after neoadjuvant therapy. Six studies did not report data on overall survival [7,9,11-14], while four studies reported an overall survival rate between 80% and 95.6% [5,6,8,10].

Disease-Free Survival Ranging From Three to Five Years

Disease-free survival was defined as the time after rectal surgery during which the tumor is not found. Six studies did not report data about disease-free survival [6,7,9,12-14]; the other four studies reported rates between 70% and 86.4% [5,8,10,11].

Urinary Disorders

Urinary disorders were defined as dysfunctions that do not present before surgery according to some scores (International Prostatic Symptoms Score (IPSS), EORTC quality of life questionnaire (QLQ)-C29). They included urinary incontinence, urinary frequency, and dysuria. Eight studies did not report data about urinary disorders [5,6,8-12,14]; two studies reported a rate between 25% and 26.5% [7,13].

Sexual Disorders

Sexual disorders were defined as dysfunction not present before surgery according to some score (Female Sexual Function Index (FSFI); International Index of Erectile Function (IIEF)). They included impotence, dyspareunia, sexual disinterest, and erectile dysfunction.

Eight studies did not report data about sexual disorders [5,6,8-12,14]. Two other studies reported a rate between 35% and 80% for both men and women [7,13].

Bowel Disorders

Bowel disorders were defined as dysfunction not present before surgery according to some score (e.g., fecal incontinence quality of life (FIQL); Wexner score; colorectal functional outcome (COREFO); low anterior resection syndrome (LARS)). They included stool frequency, fecal incontinence, abdominal pain, and flatulence.

Seven studies did not report data about bowel disorders [5,6,8,10-12,14]. Three other studies reported a rate between 17% and 44.6% [7,9,13].

Discussion

The first laparoscopic colorectal surgery was conducted in 1991, and until the beginning of the 2000s, open surgery represented the standard for colorectal surgery. With the worldwide diffusion of the laparoscopic approach, all surgeons noted its advantages, such as shorter hospital stays, fewer surgical complications, improved early postoperative quality of life, earlier postoperative bowel movements, and less pain [15]. Subsequently, debates focused on the oncological outcomes between the open and laparoscopic approaches.

According to the conventional versus laparoscopic-assisted surgery in colorectal cancer (CLASICC), COlorectal cancer Laparoscopic or Open Resection (COLOR II), and Comparison of Open versus laparoscopic surgery for mid or low REctal cancer After Neoadjuvant chemoradiotherapy (COREAN) trials, laparoscopic surgery for rectal cancer was associated with similar oncological results compared to the open approach. No differences were found in the quality of the mesorectum, circumferential and distal margins, morbidity, overall survival, disease-free survival, and local recurrence. Consequently, the laparoscopic approach seemed to be as safe as the open one, with short-term benefits. In fact, in many centers, laparoscopic surgery is used in about 80% of cases of rectal cancer [16-18].

Preliminary results of ACOSOG Z6051 and ALaCaRT trials, both published in 2015, did not show the non-inferiority of laparoscopic versus open surgery for rectal cancer. However, the subsequent results published in 2019 from these two studies showed similar quality of surgical samples with distal and circumferential margins, disease free survival and local recurrence rate between the laparoscopic and open approaches [19,20].

Transanal TME (TA TME) is an evolution of the laparoscopic technique in which, after completing the abdominal phase, the team proceeds to complete rectal mobilization through a transanal approach using specific devices to create a pneumorectum. This approach was developed to address low rectal tumors in a narrow pelvic cavity, and since 2010, it has undergone significant evolution despite the initial difficulties associated with a high rate of urethral injuries [21].

Another evolution of laparoscopic technique is transanal transection and single-stapled (TTSS) anastomosis; it is a surgical strategy developed to address low rectal cancer in a narrow pelvic cavity. It was described by Spinelli et al. in 2021 and is a very interesting technique because it is reproducible and overcomes the issues of pneumorectum and the need for special devices such as an anal port [22].

In 2000, robot-assisted laparoscopic surgery was introduced for colorectal surgery, offering advantages such as 3D optics, multiple instruments with wrists for better maneuverability, reduction of human tremor, more precise tissue dissection, and less surgical trauma. Additionally, surgeons can maintain a comfortable posture that prevents mental and physical fatigue. These factors contribute to less postoperative pain, reduced use of pain medications, and a shorter hospital stay [23].

The REAL trial suggests that robotic surgery for mid and low rectal cancer has a better oncological quality of resection compared to the conventional laparoscopic approach, based on better quality of complete resection, especially of the circumferential margin [24]. Despite the promising results from the robotic approach, the cost-benefit analysis continues to be a source of contention between surgeons and hospital managers. However, the future evolution of robotic surgery will be unstoppable [25]. Robot offers the benefit of ICG use as in laparoscopic approach, since it has an incorporated system, and it facilitates the creation of anastomoses and lateral lymph node dissection [26].

This review presents the rates of short-term oncologic outcomes, long-term outcomes, and functional outcomes. Unlike other reviews in the literature, this study emphasizes the importance of always considering both aspects when analyzing the results after rectal surgery. Furthermore, patients must be fully and accurately informed about these results when they enter a multimodal therapy program that also includes rectal surgery.

Limitations

The limitations of this review are that there are only two prospective studies, while the rest are all retrospective. We are aware of these limitations but also acknowledge that conducting prospective studies on this topic is challenging.

## Conclusions

This review demonstrates that minimally invasive surgery for mid and low rectal cancer yields good oncological and functional outcomes. Despite the higher costs, the utilization of the robotic approach is on the rise due to its short-term benefits in rectal surgery, and its ongoing development will have significant implications for rectal surgery. Further studies will be required to validate these findings.

## References

[1] Quirke P, Steele R, Monson J (2009). Effect of the plane of surgery achieved on local recurrence in patients with operable rectal cancer: a prospective study using data from the MRC CR07 and NCIC-CTG CO16 randomised clinical trial. Lancet.

[2] Kitz J, Fokas E, Beissbarth T (2018). Association of plane of total mesorectal excision with prognosis of rectal cancer: secondary analysis of the Cao/Aro/AIO-04 Phase 3 randomized clinical trial. JAMA Surg.

[3] Jayne DG, Guillou PJ, Thorpe H (2007). Randomized trial of laparoscopic-assisted resection of colorectal carcinoma: 3-year results of the UK MRC CLASICC Trial Group. J Clin Oncol.

[4] Kim HJ, Choi GS, Park JS, Park SY, Yang CS, Lee HJ (2018). The impact of robotic surgery on quality of life, urinary and sexual function following total mesorectal excision for rectal cancer: a propensity score-matched analysis with laparoscopic surgery. Colorectal Dis.

[5] Pla-Martí V, Martín-Arévalo J, Moro-Valdezate D (2021). Prognostic implications of surgical specimen quality on the oncological outcomes of open and laparoscopic surgery in mid and low rectal cancer. Langenbecks Arch Surg.

[6] Collin Å, Dahlbäck C, Folkesson J, Buchwald P (2024). Total mesorectal excision quality in rectal cancer surgery affects local recurrence rate but not distant recurrence and survival: population-based cohort study. BJS Open.

[7] Bjoern MX, Nielsen S, Perdawood SK (2019). Quality of life after surgery for rectal cancer: a comparison of functional outcomes after transanal and laparoscopic approaches. J Gastrointest Surg.

[8] Park K, Kim S, Lee HW, Bae SU, Baek SK, Jeong WK (2021). Comparison of the quality of total mesorectal excision after robotic and laparoscopic surgery for rectal cancer: a multicenter, propensity score-matched study. Korean J Clin Oncol.

[9] Pennings AJ, Vink GR, van Kuijk S, Melenhorst J, Beets GL, May AM, Breukink SO (2024). Quality of life and functional outcome of rectal cancer patients: a prospective cohort study. Colorectal Dis.

[10] Donovan KF, Lee KC, Ricardo A (2024). Functional outcomes after transanal total mesorectal excision (taTME) for rectal cancer. Results form the phase II north american multicenter prospective observational trial. Ann Surg.

[11] Peltrini R, Carannante F, Costa G (2022). Oncological outcomes of rectal cancer patients with anastomotic leakage: a multicenter case-control study. Front Surg.

[12] Francis NK, Penna M, Dritsas S (2023). Oncological outcomes after transanal total mesorectal excision for rectal cancer. Br J Surg.

[13] Shin JK, Kim HC, Lee WY (2021). Minimally invasive versus open intersphinteric resection of low rectal cancer regardless of neoadjuvant chemoradiotherapy: long-term oncological outcomes. Sci Rep.

[14] Jiang WZ, Xu JM, Xing JD (2022). Short-term outcomes of laparoscopic-assisted vs open surgery for patients with low rectal cancer. The LASRE randomized clinical trials. JAMA Oncol.

[15] Pennington E, Bell S, Hill JE (2023). Should video laryngoscopy or direct laryngoscopy be used for adults undergoing endotracheal intubation in the pre-hospital setting? A critical appraisal of a systematic review. J Paramed Pract.

[16] Jayne DG, Thorpe HC, Copeland J, Quirke P, Brown JM, Guillou PJ (2010). Five years follow-up of the medical research council CLASICC trial of laparoscopically assisted versus open surgery for colorectal cancer. Br J Surg.

[17] Van der Pas MH, Haglind E, Cuesta MA (2013). Laparoscopic versus open surgery for rectal cancer (COLOR II): short-term ouctomes of a randomised, phase 3 trial. Lancet Oncol.

[18] Jeong SY, Park JW, Nam BH (2014). Open versus laparoscopic surgery for mid-rectal or low-rectal cancer after neoadjuvant chemoradiotherapy (COREAN trial): survival outcomes of an open-label, non inferiority, randomised controlled trial. Lancet Oncol.

[19] Fleshman J, Branda ME, Sargent DJ (2019). Disease-free survival and local recurrence for laparoscopic resection compared with open resection of stage II to III rectal cancer. Follow up results of the ACOSOG Z6051 randomized controlled trial. Ann Surg.

[20] Stevenson AR, Solomon MJ, Brown CS (2019). Disease-free survival and local recurrence after laparoscopic -assisted resection or open resection for rectal cancer: the Australian laparoscopic cancer of the rectum randomized clinical trial. Ann Surg.

[21] Ng JY, Chen CC (2022). Transanal total mesorectal excision for rectal cancer: it's come a long way and here to stay. Ann Coloproctol.

[22] Spinelli A, Foppa C, Carvello M (2021). Transanal transection and single stapled anastomosis (TTSS): a comparison of anastomotic leak rates with the double-stapled technique and with transanal total mesorectal excision (TaTME) for rectal cancer. Eur J Surg Oncol.

[23] Shugaba A, Lambert JE, Bampouras TM, Nuttall HE, Gaffney CJ, Subar DA (2022). Should all mininall access surgery be robot-assisted? A systematic review into the muscoloskeletal and cognitive demands of laparoscopic and robot-assisted laparoscopic surgery. J Gastrointest Surg.

[24] Feng Q, Yuan W, Li T (2022). Robotic versus laparoscopic surgery for middle and low rectal cancer (REAL): short-term outcomes of a multicenter randomised controlled trial. Lancet Gastroenterol Hepatol.

[25] Patel SV, Wiseman V, Zhang L, MacDonald PH, Merchant SM, Barnett KW, Caycedo-Marulanda A (2022). The impact of robotic surgery on a tertiary care colorectal surgery program, an assessment of costs and short term outcomes: a Canadian perspective. Surg Endosc.

[26] Kehagias D, Lampropoulos C, Bellou A, Kehagias I (2024). The use of indocyanine green for lateral lymph node dissection in rectal cancer-preliminary data from an emerging procedure: a systematic review of the literature. Tech Coloproctol.

